# Retraction: Targeting 3-Phosphoinoside-Dependent Kinase-1 to Inhibit Insulin-Like Growth Factor-I Induced AKT and p70 S6 Kinase Activation in Breast Cancer Cells

**DOI:** 10.1371/journal.pone.0175772

**Published:** 2017-04-10

**Authors:** The PLOS ONE Editors

The author Sangita Baxi and a Pfizer representative contacted the editorial office to raise that there are image duplications in this article. Pfizer has undertaken a review and determined that there are duplications in Fig 1 and Fig 2 of the article and that part of the experiments cannot be verified.

In light of the concerns identified, the authors and the *PLOS ONE* Editors retract this article. We did not receive any response or comment from Min-Jean Yin, Wei Tan, or Tod Smeal.
